# The 4p16.3 Parkinson Disease Risk Locus Is Associated with *GAK* Expression and Genes Involved with the Synaptic Vesicle Membrane

**DOI:** 10.1371/journal.pone.0160925

**Published:** 2016-08-10

**Authors:** Michael W. Nagle, Jeanne C. Latourelle, Adam Labadorf, Alexandra Dumitriu, Tiffany C. Hadzi, Thomas G. Beach, Richard H. Myers

**Affiliations:** 1 Department of Neurology, Boston University School of Medicine, Boston, MA, 02118, United States of America; 2 Graduate Program in Molecular Translational Medicine, Boston University School of Medicine, Boston, MA, 02118, United States of America; 3 Genome Science Institute, Boston University School of Medicine, Boston, MA, 02118, United States of America; 4 Bioinformatics Program, Boston University College of Arts and Sciences, Boston, MA, 02118, United States of America; 5 Banner Sun Health Research Institute, Sun City, Arizona, 85351, United States of America; University College London Institute of Neurology, UNITED KINGDOM

## Abstract

Genome-wide association studies (GWAS) have identified the *GAK*/*DGKQ*/*IDUA* region on 4p16.3 among the top three risk loci for Parkinson’s disease (PD), but the specific gene and risk mechanism are unclear. Here, we report transcripts containing the 3’ clathrin-binding domain of *GAK* identified by RNA deep-sequencing in post-mortem human brain tissue as having increased expression in PD. Furthermore, carriers of 4p16.3 PD GWAS risk SNPs show decreased expression of one of these transcripts, *GAK25* (Gencode Transcript 009), which correlates with the expression of genes functioning in the synaptic vesicle membrane. Together, these findings provide strong evidence for *GAK* clathrin-binding- and J-domain transcripts’ influence on PD pathogenicity, and for a role for *GAK* in regulating synaptic function in PD.

## Introduction

Parkinson Disease (PD) (OMIM 168600), the second most common neurodegenerative disorder, is characterized clinically by tremor, rigidity, and bradykinesia, and neuropathologically by the presence of Lewy Bodies which are intracellular inclusions primarily composed of aggregated α-synuclein (OMIM 163890) protein. While the cellular pathogenesis of PD is not well understood, genetic studies in large sporadic and familial cohorts have revealed that heritability plays a strong role in disease risk. Recently, genome-wide association studies (GWAS) have implicated common variants at more than two dozen loci associated with increased risk for PD[1–9].

The chromosome 4p16.3 region, first identified in a GWAS of familial PD [1] and subsequently replicated[3–9], represents one of the strongest disease risk loci and contains several candidate genes, including *GAK*, *TMEM175*, *DGKQ*, *SLC26A1*, and *IDUA*. The risk allele of the GWAS SNP at 4p16.3, rs1564282[1], was significantly associated with increased mRNA expression of the α-synuclein gene *SNCA* in a microarray eSNP study of PD and control cortex[10]. This study also showed that *GAK* knockdown in the presence of over-expressed α-synuclein in HEK293 cells increased toxicity and total α-synuclein protein levels[10]. *GAK* is an essential co-chaperone along with the Hsc70 protein responsible for the dissociation of clathrin from coated intracellular vesicles during endocytosis in non-neuronal cells[11–14]. More recently, *GAK* was shown to form a protein complex with the PD-implicated protein LRRK2, which appears to be important in the trafficking of Golgi-derived vesicles through the lysosomal-autophagy system [15].

While these findings strongly suggest a role for *GAK* in PD pathogenesis, an in-depth examination of all genes in the 4p16.3 risk region to identify transcripts which are associated to GWAS risk SNPs, as well as to identify neuronal pathways in which they participate, is needed. With the advent of next generation RNA-sequencing, genes and their constitutive transcripts can now be queried for expression differences in PD brain relative to control, as well as for differences among GWAS risk SNP carriers. We therefore performed a series of RNA-seq expression studies in post-mortem brain to understand how 4p16.3 region genes and their transcripts are related to PD and to GWAS SNPS at this locus. Additionally, we modeled the expression of these significant transcripts to genome-wide expression patterns in these brains and performed pathway analysis to identify those processes which may contribute to PD pathogenesis in the context of altered *GAK* expression.

## Results

### Human Brain Sample Characteristics

We first assessed the 49 control and 29 PD human prefrontal cortical (Brodmann 9) samples assayed by RNA-sequencing for differences between cases and controls in post-mortem interval (PMI), age at death, and rs1564282 risk SNP genotype using logistic regression analysis. The age at death was found to be significantly different between cases and controls (*p*-value = 0.009), while the PMI and risk SNP minor allele frequencies (MAF) were not found to be significantly different (*p*-values = 0.092 and 0.464, respectively). The overall MAF in all samples for the rs1564282 variant was 16.0%, with 3 homozygote minor allele carriers. However, as PMI was previously reported to influence expression in post-mortem brain tissue[16,17], all association analyses were adjusted for PMI and age at death. As brain bank source is significantly associated with PMI in our samples, we did not adjust for this covariate in our analyses. Sample characteristics are described in detail in S1 Table.

### *GAK* Gene Expression is Increased in Parkinson Disease

We analyzed the expression of genes in the 4p16.3 region (containing *GAK*, *TMEM175*, *DGKQ*, *SLC26A1*, and *IDUA*) for association to case status in all samples using a linear regression analysis. We found only *GAK* to be significantly increased in expression in PD after adjusting for multiple comparisons (β-estimate = 0.29, *q*-value = 4.8x10^-9^). We further examined the expression of these genes for association to the PD GWAS risk SNP rs1564282 using linear regression analysis, adjusting for case status, but found no significant association between gene expression and the risk allele.

### Gencode-annotated Exon Feature Expression in the 3’ Region of *GAK* is Associated with Case Status and Risk SNP rs1564282

**Fig 1:**
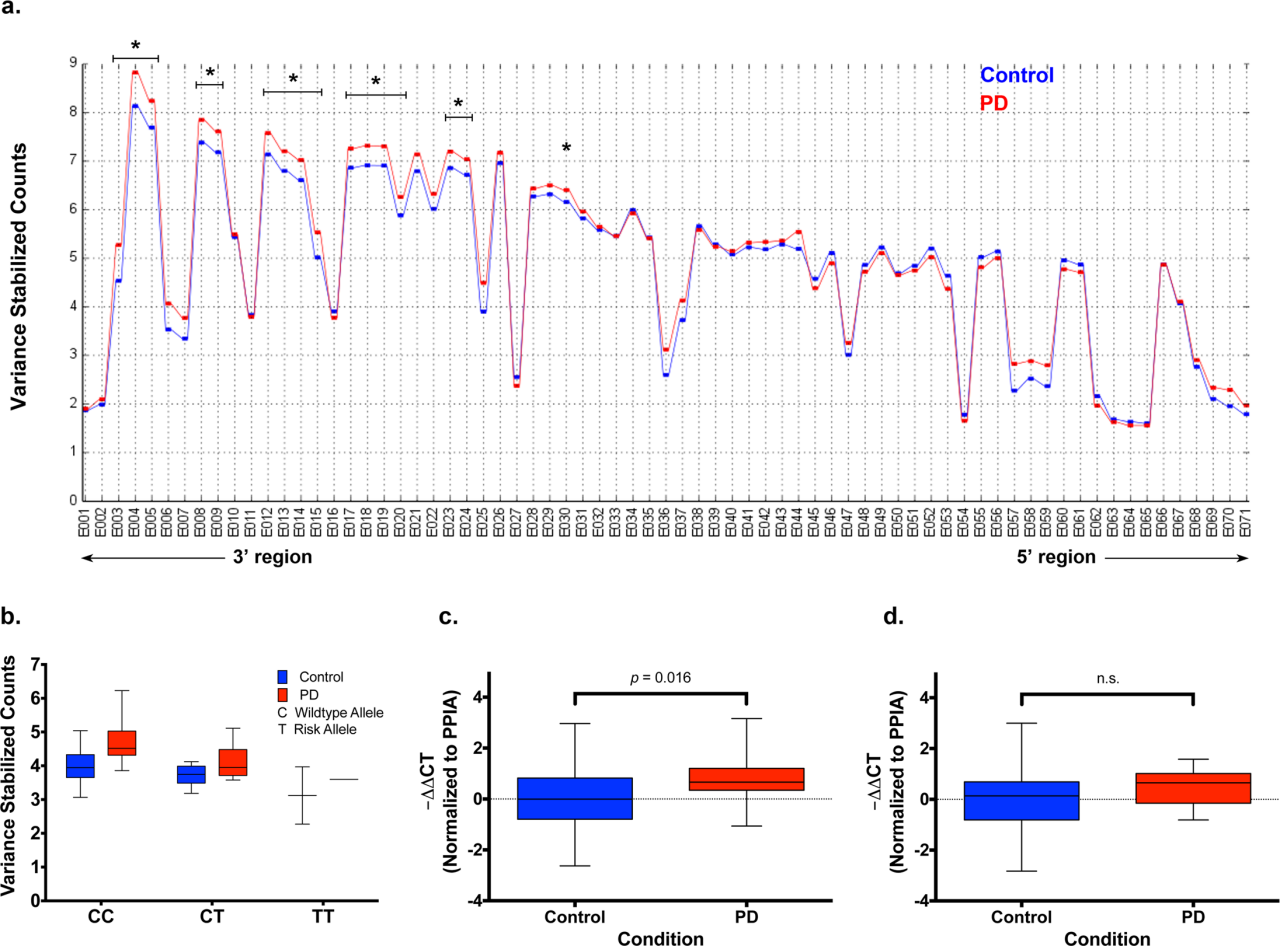
*GAK* Gencode exons in the 3’ region are differentially expressed in PD. (A) The mean normalized expression for PD cases and controls is plotted for each of the 71 *GAK* exon feature bins (x-axis). Exon 1 corresponds to the 3’ end of the gene, while Exon 71 corresponds to the 5’ end. Brackets indicate groups of exons significantly increased in PD relative to control (* = *q*-value < 0.05). (B) The mean normalized expression of *GAK* exon 25 (*GAK25*) is plotted for each stratified group. The samples are stratified by case status (Control and PD) and by genotype in an additive fashion (CC = Major allele homozygote, CT = Heterozygote, TT = Risk allele homozygote). (C and D) -ΔΔCT (log2 fold change) of *GAK* normalized to *PPIA* is plotted for all control and PD samples for (**c**) *GAK* 3’ exons and (**d**) *GAK* 5’ exons.

In order to assess association of 4p16.3 exon features’ expression to the risk SNP rs1564282, we performed a multivariate linear regression analysis, adjusting for age at death, PMI, and case status. Out of 207 exon features (as annotated by the Gencode version 14 gene annotation) assessed in the five genes at this locus, we found only one exon feature, *GAK* exon 25 (hereafter referred to as *GAK25*), to be significantly associated to both case status and the risk SNP in the analysis after adjusting for multiple comparisons. In further analysis of *GAK*, we evaluated its exon features’ expression for association to case status utilizing a linear regression analysis. We found twenty-four out of seventy-one exon features in *GAK* had FDR-significant increased expression at FDR-level of significance in PD relative to control (Fig 1A); additionally, none of the exon features in *GAK* showed significantly decreased expression in PD, and those that are increased in PD are exclusively located in the 3’ region of the gene (Fig 1A). Intriguingly, *GAK25* expression was increased in PD relative to controls (β-estimate = 0.54, *q*-value = 3.3x10^-4^), but was decreased in risk SNP carriers (both PD and control SNP carriers) relative to non-risk SNP carriers (β-estimate = -0.40, *q*-value = 0.035) (Fig 1B).

In order to validate the findings of increased expression of the 3’ region of *GAK* in PD from the RNA-sequencing study, we performed qPCR analysis on the same samples using primer-probe sets that target the exon-junctions between Gencode exons 5 and 8 (3’ exons) and Gencode exons 61 and 66 (5’ exons). We found expression to be increased in PD relative to control in the 3’ region of *GAK* (β-estimate = 0.67, *p*-value = 0.016) (Fig 1C), but found no significant difference between case and control in the 5’ region of *GAK* (Fig 1D) after adjusting for age at death and PMI.

### Expression of *GAK25* Strongly Correlates with Expression of 3’ *GAK* Exons

**Fig 2:**
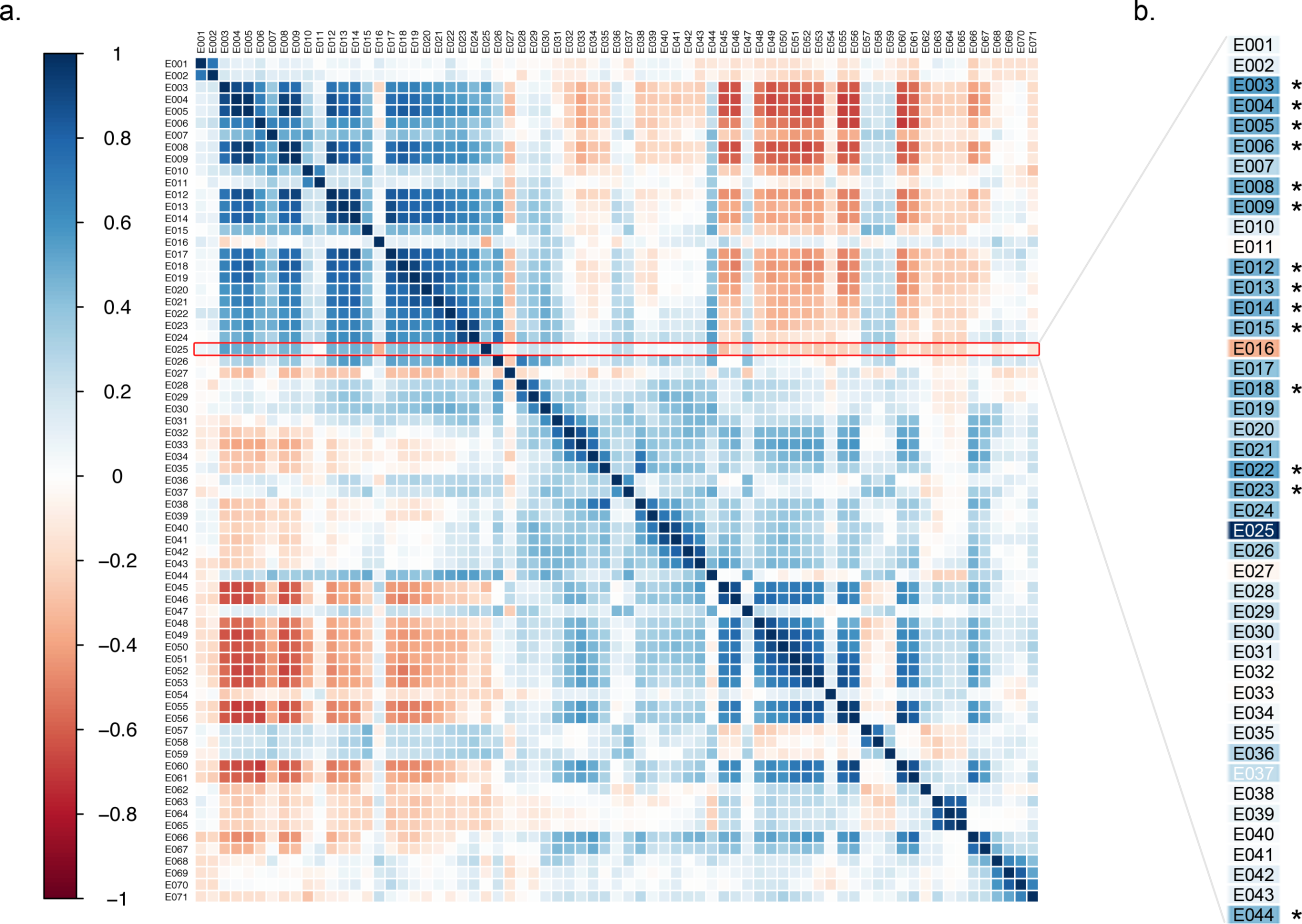
*GAK* Gencode 3’ exons are significantly correlated with *GAK* Gencode exon 25 by Spearman correlation. (A) The numbers along the color scale represent the r-values for each correlation. Positive correlations are represented by blue shades, while negative correlations are represented by red shades. The 3’ end of the gene corresponds to exon one (E001 in the upper left-hand corner), while the 5’ end of the gene corresponds to E071. (B) Exons significantly associated with *GAK25* (using an adjusted α-level of 1.6x10^-4^) are starred and are found predominantly in 3’ region. Exons 1–44 are shown, as the exons contained in this range were the only ones significantly correlated after correction for multiple comparisons.

As the exons in the 3’ region of *GAK* are constituents of larger transcripts, the expression of *GAK25* is not independent of the expression of these other 3’ exons. In order to better elucidate the expression patterns of the exons in *GAK* and how they relate to one another, we performed a Spearman correlation analysis among all the *GAK* exons. We found high correlation among the exons features in the 3’ region of the gene (Fig 2A). While the correlation was equally strong among exon features in the 5’ region of the gene, the two regions were not strongly correlated with each other (Fig 2A). Furthermore, when we examined the correlation between *GAK25* and other *GAK* exon features, we found significant and strong correlation between *GAK25* and 3’ exon features in *GAK*, particularly those which are associated with case status (Fig 2B), with *p*-values less than the multiple comparison adjusted α-level of 1.6x10^-4^.

### Expression of *GAK25* is Associated with Differential Transcriptomic Expression

We next performed analyses to identify global patterns of transcriptomic expression associated with *GAK25*, and by extension 3’ *GAK* exons expression in PD, in order to identify gene sets and pathways related to dys-regulated *GAK* expression. Using all cases and controls, regardless of risk allele carrier status, we assessed the association of *GAK25* expression to all genes (utilizing the pre-filtered, batch-adjusted, and variance-stabilized gene counts) using linear regressions adjusting for age at death and PMI. Of the 23,676 genes assessed, we found 788 genes associated with *GAK25* at Bonferroni threshold for multiple comparison (α = 2.11E-06). Of these associated genes, 562 were inversely associated with *GAK25* expression, while 226 were directly associated. The full list of these genes can be found in S2 Table.

### Pathway Analysis Reveals *GAK*’s Influence on Genes Involved in Synaptic Structure, Nucleoside Phosphate Binding, and Mitochondrial Function

In order to determine the functional relevance of the gene sets associated with *GAK25* expression, we performed GO term enrichment analysis of the genes significantly associated to *GAK25* using the topGO package implemented in R. Out of the 23,676 total genes assessed in the RNA-seq study, 14,754 were functionally annotated and 557 of these were significantly associated to *GAK25*. We utilized the classic algorithm implemented in the package to identify the most significantly enriched nodes, and found 21 GO terms significantly enriched at Bonferroni corrected *p*-value less than 0.05 (Table 1). These include gene-sets particularly related to synaptic and mitochondrial structure. As many of these nodes contain overlapping genes, and are highly correlated in their common gene membership, we sought to identify the most important nodes among them. We therefore utilized the weight algorithm implemented in the topGO package, which identifies the most significantly enriched local nodes within the GO graph given its structure, and found only one term, synaptic vesicle membrane, to be significantly enriched after Bonferroni correction (α = 0.05) (Table 1).

**Table 1:** Genes Associated with *GAK25* Expression in PD brain are Significantly Enriched in GO Terms Related to Synaptic and Mitochondrial Structure.

GO Term ID	GO Term Description	Annotated Genes	Significant Genes	Classic Algorithm (Bonferroni-corrected)	Weighted Algorithm (Bonferroni-corrected)
GO:0030672	synaptic vesicle membrane	54	14	1.43E-05	1.43E-05
GO:0005739	mitochondrion	1537	103	1.15E-04	0.50836
GO:0045202	synapse	642	53	4.65E-04	1
GO:0008021	synaptic vesicle	121	18	1.29E-03	1
GO:0044446	intracellular organelle part	7129	344	1.86E-03	1
GO:0031966	mitochondrial membrane	607	49	2.36E-03	1
GO:0044422	organelle part	7307	349	4.30E-03	1
GO:0044456	synapse part	516	43	4.30E-03	1
GO:0031090	organelle membrane	2789	156	4.65E-03	1
GO:0005740	mitochondrial envelope	643	50	5.30E-03	1
GO:0044444	cytoplasmic part	7191	343	7.09E-03	1
GO:0097458	neuron part	1110	74	0.010024	1
GO:0098793	presynapse	141	18	0.011456	1
GO:0043231	intracellular membrane-bounded organelle	9475	431	0.013604	1
GO:0017111	nucleoside-triphosphatase activity	729	54	0.013767	1
GO:0043234	protein complex	3722	194	0.022912	1
GO:0036094	small molecule binding	2415	136	0.023298	1
GO:0044429	mitochondrial part	851	59	0.025776	1
GO:0016817	hydrolase activity, acting on acid anhydrides	770	55	0.03177	1
GO:0000166	nucleotide binding	2160	123	0.039183	1
GO:1901265	nucleoside phosphate binding	2161	123	0.040242	1

## Discussion

Using an agnostic region-wide approach, we found PD GWAS SNP risk alleles in the 4p16.3 region to be associated with significantly decreased expression of the Gencode defined exon 25 (*q* = 0.035) of the *GAK* gene (*GAK25*). Additionally, exons in the 3’ region of *GAK* were increased in expression in PD relative to controls in human prefrontal cortical brain tissue. Finally, we observed genome-wide significant associations of *GAK25* in the RNA-sequencing analysis with the expression of several genes functioning at the synapse and mitochondria suggesting co-regulation of expression of these genes in cortical tissue. Together, these results support the attribution of the observed PD GWAS signal at 4p16.3 to *GAK* and provide novel interactions and roles for this protein in the pathogenesis of PD.

**Fig 3:**
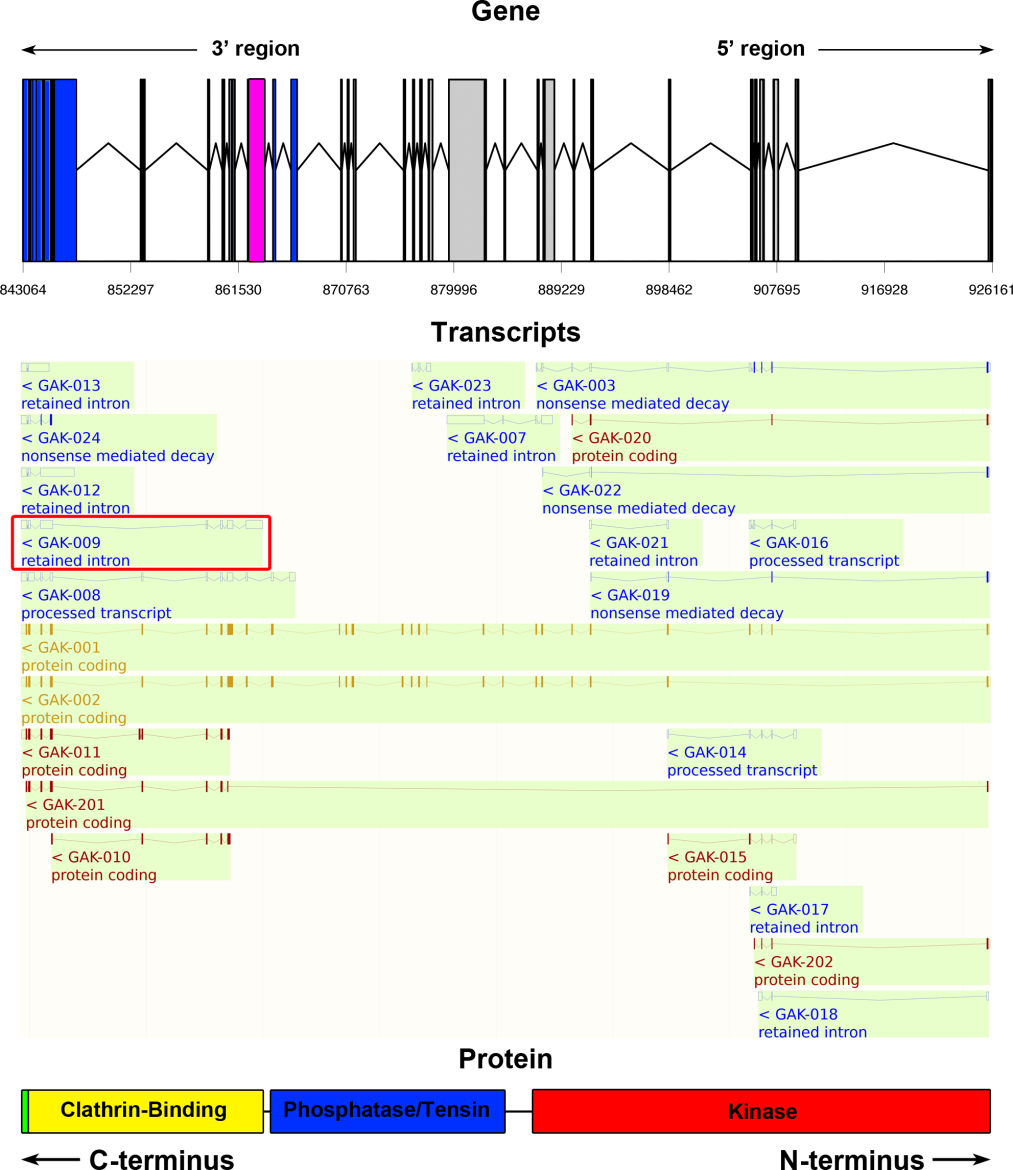
*GAK* exons significantly associated to case status primarily encode for the clathrin-binding and J domains of *GAK* protein. *GAK* exon 25 (*GAK25)*, which was found to be associated to both case status and the risk SNP, is highlighted in magenta (under Gene), while *GAK* exons associated with case status only are highlighted in blue. All Gencode transcripts are shown below the gene structure, with transcript functionality listed below each contig. *GAK25* represents the 5’UTR of Gencode transcript 009 (Boxed in red). The *GAK* protein domains (under Protein) are denoted by color: J domain (green), clathrin-binding domain (yellow), phosphatase/tensin domain (blue), and kinase domain (red). Transcript data from http://www.ensembl.org.

The Gencode project has identified several protein-coding transcripts in the 3’ region of *GAK*, including one that utilizes *GAK25* as the 5’ UTR (Transcript 009, Fig 3). As *GAK25* is uniquely associated with this single transcript, we hypothesize that this transcript is also increased in expression in PD. Furthermore, *GAK25* is highly correlated with the expression of the other 3’ exons in *GAK*, and as many of these other 3’ exons are expressed in higher amounts relative to *GAK25*, this suggests that other 3’ truncated transcripts may also be increased in PD. Because these smaller transcripts hypothetically translate to form proteins containing only the clathrin-binding and J domains of GAK, as indicated by the Ensembl project, it appears these abbreviated proteins (009, 010, 011, and 201 in Fig 3) may have an important role in mitigating the effect of the disease, discussed below, possibly through the function of their domains in facilitating clathrin-mediated endocytosis[11,13,14,18].

The observation that exons in the 5’ region of *GAK* encoding the kinase and phosphatase/tensin domains of the protein are not differentially expressed in PD suggests that 5’ abbreviated transcripts (003, 007, and 014 through 023 in Fig 3), as well as the full-length transcripts (001 and 002 in Fig 3) of *GAK* may have little functional relevance to PD risk. Although the read depth at a few of these 5’ exons is low relative to 3’ exon expression, several exons in the center of the gene, which constitute both the 5’ and full length transcripts, have reasonable read depth, and are not differentially expressed in PD (Fig 3 and Fig 1A). Although 3’ exons of *GAK* showed increased expression in PD relative to controls, the GWAS SNP risk allele was associated with reduced expression. Specifically, reduced *GAK25* was significantly associated with both case status and the risk SNP in a multivariate linear regression after FDR adjustment. As the minor allele of the risk SNP has been shown to increase the risk for PD, we hypothesize that reduced *GAK25* and 3’ exon expression may represent a compromise of an adaptive and potentially protective response of the abbreviated 3’ transcripts of *GAK* to the disease.

Based on canonical pathway analysis and functional annotation of the genes associated to *GAK25* in the RNA-sequencing analysis, *GAK* appears to have a significant relationship to genes playing a role in synaptic and mitochondrial structure in post-mortem brain. As clathrin-mediated endocytosis (CME) plays a crucial role at the pre-synaptic terminal in recycling vesicles and at the post-synaptic terminal in regulating receptor signaling, and *GAK* has previously been shown to play a crucial role in CME, we hypothesized that *GAK* expression would be associated with genes having a functional role in CME or pathways related to this function. Indeed, *GAK25*-associated genes are enriched in several GO terms related to the synaptic vesicle membrane (Table 1), demonstrating a specific connection between *GAK* and synaptic structural components. Moreover, the significant genes playing a role in synaptic vesicle membrane structure, including synaptotagmins I and XII (*SYT1* and *SYT12*), vesicular glutamate transporter (*SLC17A8*), and GABA vesicular transporter (*SLC32A1*), were all inversely associated with *GAK25*; this association suggests *GAK*’s up-regulation in PD may be functionally related to these genes’ down-regulation and synaptic dysfunction. As synaptic function has been previously observed in many studies to be negatively affected in PD[19–21], this observation provides novel evidence for *GAK*’s role in maintaining proper function at the synapse, with dysfunctional synaptic function occurring concurrently with *GAK* differential expression in PD.

Additionally, we observed significant functional enrichment of *GAK25*-associated genes annotated for mitochondrial structure. This finding potentially connects aberrant *GAK* expression to pathways that, when dys-regulated through chemical induction via MPTP catabolism[22] or monogenic loss-of-function of essential mitochondrial genes[23–26], have been previously observed to cause early-onset parkinsonism. In particular, *GAK25* was inversely associated with the *PARK2* gene, which codes for the parkin protein and functions as an E3 ligase[27] important in the maintenance of mitochondrial integrity through mitophagy[28,29]. As *GAK* has been previously shown to play a role in autophagy through its interaction with the PD-associated gene *LRRK2*[15], this novel finding relating *GAK* to impaired mitochondrial function and potentially to impaired mitophagy provides an additional mechanism through which *GAK* risk SNPs might mediate their effect.

While this study demonstrates strong associations between risk variants in the 4p16.3 locus and 3’ transcripts of *GAK*, as well as elucidates potential mechanisms through which *GAK* might mediate its effects, the causal relationship between *GAK* and these pathogenic mechanisms has yet to be demonstrated. Fine-mapping studies combined with complementary multi-omic approaches to identify the causal risk variant in the *GAK* locus driving the genetic effect, as well as studies evaluating the effect of dys-regulated expression of 3’ transcripts of *GAK* in model systems, are warranted. As *GAK* is ubiquitously expressed, it will be particularly important to evaluate the effect of *GAK* 3’ transcript dys-regulation in the context of the neuronal and glial systems that have previously been evaluated to understand PD pathogenetic mechanisms.

In summary, prefrontal cortical expression of 3’ exons in *GAK* is associated not only with PD risk and the GWAS SNP rs1564282, but also with the expression of genes regulating synaptic vesicular membrane structure and mitochondrial structure. Dysfunctional regulation of *GAK* in those PD patients who carry the 4p16.3 GWAS risk variant appears to be related to gene transcription related to synaptic and mitochondrial biology, which would drastically affect the neuron’s ability to function properly, especially while under the burden of the aggregated α-synuclein protein. Through these analytical approaches, we have provided in-depth investigation into pathways affected by altered *GAK* expression, which may provide us with future targets for therapeutic intervention in PD.

## Methods

### Brains Studied for RNA-sequencing

Cryo-preserved post-mortem Brodmann Area 9 prefrontal brain tissue from PD cases with no extensive evidence of AD pathology and from neuropathologically-confirmed controls was acquired from three different brain banks: the Harvard Brain Tissue Resource Center at McLean Hospital (Belmont, Massachusetts), the Human Brain and Spinal Fluid Resource Center at VA West Los Angeles Healthcare Center (Los Angeles, California) and the National Brain and Tissue Resource for Parkinson’s Disease and Related Disorders at Banner Sun Health Research Institute[30] (Sun City, Arizona). All samples used in the RNA-sequencing study were male in gender and the donors were all Caucasian. A description of the samples studied can be found in S1 Table.

### Total RNA Collection and Quality Control from Human Tissue

Approximately 0.1g of grey matter tissue from each sample section was used for total RNA extraction using the Qiagen miRNeasy Mini Kit (Hilden, Germany) following the standard protocol from the manufacturer. Total RNA was quantified by 260nm OD measurement on a Biotek (Winooski, Vermont) Synergy H1 Hybrid Plate reader using a Take3 Micro-Volume Plate. RNA quality for each sample was assessed with the Agilent Technologies (Santa Clara, California) 2100 Bioanalyzer using standard protocol as specified by the manufacturer. Samples with RNA Integrity Numbers (RINs) greater than 6.0 were used in the studies and samples failing to reach this threshold were re-extracted or excluded. RNA quality assessment details for each sample can be found in S1 Table.

### cDNA Library Preparation and Sequencing

Libraries of cDNA derived from poly-adenylated mRNA were created at the Tufts University Sequencing Core Facility (Boston MA, http://tucf-genomics.tufts.edu/) from the total RNA for each sample using the Illumina (San Diego, California) TruSeq mRNA Sample Preparation Kit following the manufacturer’s protocol. We utilized 1 ug of RNA total for the library preparation of each sample at a concentration of 100 ng/uL. Libraries were indexed and pooled for multiplex sequencing on an Illumina HiSeq 2000 System at the Tufts University Sequencing Core Facility. Five separate sequencing batches were performed in order to obtain data for all samples; the batch run for each sample is indicated in S1 Table. Sequence reads were specified to be paired-end (sequenced from both ends of the cDNA fragment) and approximately 100 bp long. The sequence data was demultiplexed and output to FASTQ files for each sample using the Illumina Consensus Assessment of Sequence and Variation (CASAVA) pipeline (with two files generated per file corresponding to each end read), which were then used for subsequent analysis. Data from these experiments are publicly available (GEO accession numbers GSE68719 and GSE64810).

### Data Alignment and Quality Control Analysis

Sequence data were aligned to the UCSC human reference genome 19 using the spliced read mapper Tophat version 2.0.6[31], which first aligns short reads using the high-throughput aligner Bowtie version 2.1.0[32], followed by analysis of the mapped results for the presence of splice junctions. Read inner distance (the length of unsequenced fragment cDNA between paired reads) and standard deviation of the inner distance were estimated to be 50 bp each (the—mate-inner-dist option and—mate-std-dev option, respectively). The number of mismatches to the genome allowed for each read was 3 (—read-mismatches option) while the number of splice mismatches allowed was 1 (—splice-mismatches option), to account for the presence of single nucleotide polymorphisms. Finally, the read edit distance (—read-edit-dist option) was set to 3 to account for the possibility of in-del mutations, and the maximum number of alignments to the genome for each read (—max-multihits option) was set to 20. All other alignment options were set to default.

The output BAM files from the alignment were then used to perform quality control analysis using the RSeQC python package[33]. This package assesses reads mapping statistics (through the bam_stat.py script), including the total number of aligned reads from overall total reads, how many of those reads have a matched pair for a particular fragment, and how many of the reads map to splice junctions versus non-spliced sequences. It can also assess the gene body coverage (geneBody_coverage.py), GC content of each read (read_GC.py), and read quality according to each read’s requisite Phred score (read_quality.py) among other assessment options. None of the samples were assessed as outliers among the group as evaluated by all the parameters measured using RSeQC, and so all were carried forward in the analysis.

### mRNA Gene and Exon Abundance Quantification

The alignment BAM files for each sample were first sorted by read names and had reads extracted from the binary BAM format to the SAM format using SAMtools[34]. Using the GENCODE version 14 annotation GTF file (http://www.gencodegenes.org/releases), raw counts were extracted for each gene from the sorted SAM file of each sample using the htseq-count function implemented in HTSeq version 0.5.3p9 (http://www-huber.embl.de/users/anders/HTSeq). Next, an annotation GFF file containing collapsed exon/partial exon counting bins with specific boundaries for all known human transcripts was created from the GENCODE GTF file utilizing the DEXSeq python function dexseq_prepare_annotation.py[35]; this new exon annotation file, along with the sorted SAM file for each sample, were used to count paired reads in each exon counting bin using the DEXSeq script dexseq_count.py implemented through HTSeq.

### Gene and Exon Expression Normalization and Gene Specific Exon Analysis

The genome-wide gene raw read counts for each sample were next normalized in a counts data set object using the estimateSizeFactors function implemented in the R (http://www.R-project.org) package DESeq2 version 1.4.5[36,37]. Genes with mean normalized counts in both cases and controls less than two were filtered prior to further analysis. The normalized counts were then transformed to stabilize the variances and create a more normal distribution using the varianceStabilizingTransformation function in DESeq2; this was followed by performing correction for sequencing batch for the samples using the ComBat function in the R package sva version 3.8.0[38,39]. The resulting batch-corrected and variance-stabilized gene counts object was used to extract counts for genes in the 4p16.3 region (which include *GAK*, *TMEM175*, *DGKQ*, *SLC26A1*, and *IDUA*) based on their proximity to the PD risk SNPs in the region; these counts were used for region-specific analysis. In a similar fashion, the genome-wide exon feature raw read counts for each sample were normalized in DESeq2 using the same function, followed by variance stabilization and batch adjustment. Exon-specific variance-stabilized and batch-corrected counts were then extracted for genes in the chromosome 4p16.3 region and used for downstream analysis.

Genes in 4p16.3 were assessed for their differential expression in all cases relative to all controls using linear regression analysis (glm function in R) adjusting for age at death and post-mortem interval (PMI). The resulting *p*-values for these regional analyses were adjusted for multiple comparisons using the false discovery rate (FDR) control (*q*-value) as described by Benjamini and Hochberg[40], with a threshold cutoff of 0.05. A multivariate linear regression analysis was then used to model the association of 4p16.3 genes’ expression to case status and the risk SNP rs1564282 previously genotyped in these brains[10] (in an additive genetic model), adjusting for age at death and PMI.

In a similar fashion, the exon features of genes in the 4p16.3 region were assessed for their differential usage in all cases relative to all controls using linear regression adjusting for age at death and PMI. Additionally, a multivariate linear regression was used to model the association of 4p16.3 exon features’ expression to case status and the risk SNP rs1564282, adjusting for age at death and PMI. Next, a Spearman correlation analysis was performed between the exon features in *GAK* to assess the correspondence in expression among them. To account for 2,485 comparisons (unique correlations between all 71 exon features of *GAK*), an adjusted α-level of significance of 1.6x10^-4^ was used; this was obtained by applying a modified Bonferroni correction[41] which takes into account the degree of correlation among the exons (mean *R* = 0.26).

Finally, we assessed genes for their association to *GAK25* expression using the variance stabilized batch adjusted counts for genes and for *GAK* exon feature 25 using a linear regression analysis, adjusting for age and PMI, and evaluated for downstream functional annotation only those genes which showed significant association at the Bonferroni adjusted cut-off of α = 2.11E-06.

### Comparative, Functional, and Pathway Analyses

The list of genes significantly associated to *GAK25* were functionally annotated using gene ontology (GO) term enrichment via the R package topGO[42] and the genome-wide annotation for Human database[43]. Biological Processes (BP), Molecular Functions (MF), and Cellular Component (CC) terms were assessed for enrichment independently using the classical Fisher enrichment method, adjusting for multiple comparisons within each GO term group. We additionally evaluated the significant genes for enrichment in these same GO terms using the weight method[42,44], which assesses connected nodes within the GO graph in order to detect the locally most significant terms. We utilized default options for both analyses, and GO terms were deemed significant if their Bonferroni-adjusted *p*-values were less than 0.05 using either enrichment method.

## Supporting Information

S1 Table Descriptive Characteristics of Brain Samples Studied. (XLSX)

S2 Table Genome-wide association to GAK25 Expression. Ensembl ID and Gene Symbol are provided for each gene. Estimate represents the effect estimate for association after adjustment for PMI and Age of Death. The Bonferroni-adjusted threshold for significant association is α = 2.11E-06.
(XLSX)
